# Intelectin-1 is a novel prognostic biomarker for hepatocellular carcinoma

**DOI:** 10.1097/MD.0000000000036474

**Published:** 2023-12-01

**Authors:** Jiang Li, Hai-Su Tao, Tong Yuan, Zhi-Yong Huang, Er-Lei Zhang

**Affiliations:** a Hepatic Surgery Center, Tongji Hospital, Tongji Medical College, Huazhong University of Science and Technology, Wuhan, China; b Department of Hepatobiliary Surgery, The First Affiliated Hospital, Shihezi University, Shihezi, Xinjiang, China.

**Keywords:** hepatocellular carcinoma, inteltin-1, prognosis, survival

## Abstract

The molecular mechanisms of hepatocellular carcinoma (HCC) are still not well understood. Gene microarray analysis showed that the expression of Intelectin-1 (ITLN-1) in tumor-adjacent normal liver tissue was 454.8 times higher than in the corresponding cancer tissue. ITLN-1 is a secreted soluble glycoprotein which has been reported to be associated with the occurrence and development of various tumor types. However, the prognostic significance of ITLN-1 in HCC remain unclear. Real-time fluorescence quantitative polymerase chain reaction was used to investigate 149 liver cancer cases for ITLN-1 mRNA expression. Immunohistochemistry and western blot analysis were used to ascertain protein expression of ITLN-1 in cancer and para-carcinomatous tissue, and further to evaluate the correlation between ITLN-1 mRNA expression and surgical prognosis after liver resection. The ITLN-1 mRNA and protein levels were significantly higher in adjacent normal liver tissues than HCC tissues. Real-time fluorescence quantitative polymerase chain reaction showed that the ITLN-1 expression was decreased in 78.5% (117/149) of HCC tissues compared with their corresponding adjacent liver tissues. Moreover, its low expression was significantly correlated with increased tumor size, tumor differentiation degree, degree of liver cirrhosis, capsule integrity, vascular invasion and tumor recurrence. Patients with high ITLN-1 expression had significantly better overall and recurrence-free survival after curative liver resection. Multivariate cox regression analysis showed that ITLN-1 was an independent predictor of surgical outcomes in HCC patients. The present study suggested that low ITLN-1 expression was associated with poor clinical outcome for HCC patients, indicating a novel biomarker for prognosis evaluation and a potential therapeutic target for HCC patients.

## 1. Introduction

Hepatocellular carcinoma (HCC) is one of the most common malignancies in the world. In 2018, there were 841,080 new cases of liver cancer worldwide, ranking seventh in morbidity, as well as 781,631 deaths, ranking third in mortality among all malignancies.^[1]^ China is a high-incidence area of HCC, accounting for about half of new cases in the world each year, with HCC ranking fourth in morbidity and third in mortality among all malignant tumors.^[2]^ Hepatectomy is still the most important treatment for HCC in China. However, the overall efficacy of hepatectomy is still poor, with a 5-year total recurrence rate after hepatectomy as high as 80%, and 5-year overall survival (OS) of only 30% to 70%.^[3,4]^ Postoperative recurrence, intrahepatic metastasis and insensitivity to chemotherapy are important factors affecting the long-term outcomes of hepatectomy. During the pathogenesis of HCC, the expression of thousands of genes is dysregulated. The abnormally high expression of oncogenes and the inactivation of tumor suppressor genes are key drivers of the occurrence and development of tumors.^[5]^ Therefore, the search for new abnormally expressed molecular markers related to the occurrence and development of HCC may contribute to the diagnosis and treatment of HCC.

**Figure 1.**
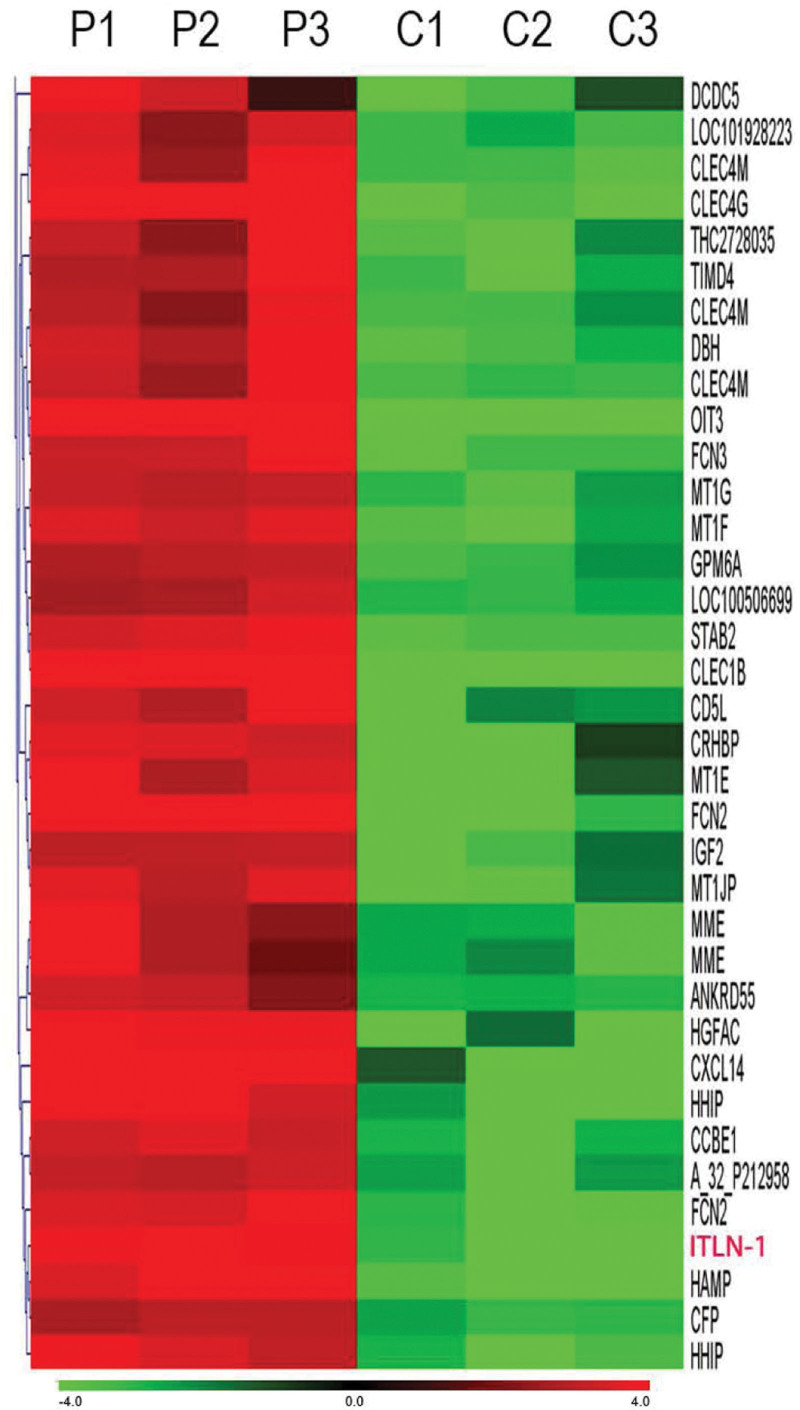
The expression of ITLN-1 was 454.8 times higher in tumor-adjacent normal liver tissues than in corresponding cancer tissues.

Although great efforts have been made to elucidate the pathogeny, but the detailed molecular mechanisms of HCC are still not well understood. In order to identify the candidate genes in the carcinogenesis and progression of HCC, and provide the basis for the prevention and treatment of HCC, we used microarray analysis to screen out intelectin-1 (ITLN-1) and for our further study. We previously used gene chip technology to detect differentially expressed genes in 3 pairs of liver cancer tissues and tumor-adjacent normal liver tissues, and screened out 30 differentially expressed genes whose expression was more than 50 times higher in tumor-adjacent normal liver tissues than in the corresponding cancer tissues, which may have the function of predicting the development of HCC. The expression of ITLN-1 was 454.8 times higher in tumor-adjacent normal liver tissues than in corresponding cancer tissues (Table 1 and Fig. 1). Therefore, ITLN-1 may be a potential new molecular marker for detection of HCC.

**Table 1:** 30 molecules with more than 50-fold difference in expression between liver cancer tissues and peri-cancer liver tissues.

Gene	Description	Fold
CRHBP	Corticotropin releasing hormone binding protein	911.5
MT1G	Metallothionein 1G	726.8
ITLN-1	Intelectin 1	454.8
MME	Membrane metallo-endopeptidase	413.9
STAB2	Stabilin 2	195.3
IGF2	Insulin-like growth factor 2 (somatomedin A)	192.4
HAMP	Hepcidin antimicrobial peptide	182.3
CCBE1	Collagen and calcium binding EGF domains 1	133.01
OIT3	Oncoprotein induced transcript 3	120.1
CLEC4	C-type lectin domain family 4, member M	113.4
MT1F	Metallothionein 1F	105.8
DBH	Dopamine beta-hydroxylase	99.9
FCN3	Ficolin (collagen/fibrinogen domain containing) 3	93.6
FCN2	Ficolin (collagen/fibrinogen domain containing lectin) 2	92.4
CFP	Complement factor properdin	86.9
DCDC5	Doublecortin domain containing 5	81.6
CD5L	CD5 molecule-like	71.8
TIMD4	T-cell immunoglobulin and mucin domain containing 4	71.8
CXCL14	Chemokine (C-X-C motif) ligand 14	71.8
CLEC4	C-type lectin domain family 4, member M	65.3
HHIP	Hedgehog interacting protein	62.1
MT1E	Metallothionein 1E	60.9
FCN2	Ficolin (collagen/fibrinogen domain containing lectin) 2	57.8
GPM6A	Glycoprotein M6A	56.0
CLEC4G	C-type lectin domain family 4, member G	55.5
CLEC1B	C-type lectin domain family 1, member B	55.2
ANKRD	Ankyrin repeat domain 55	54.7
HGFAC	HGF activator	54.7
MT1JP	Metallothionein 1J, pseudogene	53.7
HHIP	Hedgehog interacting protein	52.9

ITLN is a secreted soluble glycoprotein that belongs to the animal lectin family encoded by the ITLN-1 and ITLN-2 genes. Studies have shown that ITLN-1 has antimicrobial, antiinflammatory, and immunomodulatory effects,^[6]^ which has been reported to be associated with the occurrence and development of various tumor types. The expression of ITLN-1 in the gastric mucosa of patients with gastric carcinoma is significantly increased, and the expression level of ITLN-1 is significantly correlated with tumor differentiation, the depth of tumor invasion into the gastric wall, lymph node metastasis, distant metastasis, and clinical stage of gastric cancer, suggesting that ITLN-1 is an independent prognostic factor related to the long-term survival of patients with gastric cancer.^[7]^ A high concentration of ITLN-1 was detected in the pleural effusion of patients with malignant pleural mesothelioma,^[8]^ suggesting that malignant pleural mesothelioma cells can secrete ITLN-1, and it is believed that ITLN-1 can be used as a biomarker for the diagnosis of malignant pleural mesothelioma. However, the role and specific mechanisms of ITLN-1 in the development of malignant pleural mesothelioma are still unclear.^[9,10]^ Other studies have shown that ITLN-1 can inhibit tumor progression and play the role of a tumor suppressor gene in neuroblastoma,^[11]^ gastric cancer^[12]^ and colon cancer.^[13,14]^ Therefore, the role of ITLN-1 in different types of tumors remains controversial.

The purpose of this study was to identify the role of ITLN-1 in HCC, and to explore the relationship between ITLN-1 and clinicopathological characteristics of HCC patients, as well as the significance of ITLN-1 expression in the prognosis of HCC.

## 2. Materials and methods

### 2.1. Patients and samples

For this study, we collected tumor tissues and matched non-tumor liver tissues from 149 HCC patients admitted to the Department of Blinded for peer review between January 2013 and December 2014. All samples were shock-frozen in liquid nitrogen and stored at −80 °C immediately after surgical resection. According to the clinical diagnosis and staging criteria of primary liver cancer, patients meeting all of the following eligibility criteria were included in our study: (1) diagnosed with primary liver cancer by histopathological examination; (2) radical resection; (3) complete follow-up data; (4) no preoperative radiotherapy, chemotherapy, immunotherapy or targeted molecular therapy; (5) no family history of malignant tumors or malignant tumor of other organs; (6) there was no death within 3 months after surgery. Follow-up data were as follows: serum alpha-fetoprotein (AFP) levels were monitored every 2 months and ultrasonography was performed. Chest radiographs were taken every 6 months in the first 2 years after surgery and every 3 to 6 months thereafter. Patients with abnormal AFP or ultrasound results were examined by computed tomography or magnetic resonance imaging. Baseline patient information was obtained from the electronic medical record system, including age, sex, family history, hepatitis B surface antigen, AFP, cirrhosis, tumor size and number, vascular invasion, Barcelona Clinic Liver Cancer staging, metastasis and recurrence, degree of pathological differentiation, tumor-node-metastasis (TNM) stage, and capsule status. According to the 7th TNM Staging of the International Union for Cancer Control and the American Joint Committee on Cancer,^[15]^ OS was defined as the time from surgery to the patient death or last follow-up. Disease-free survival (DFS) was defined as the time from surgery to recurrence or distant metastasis of HCC. This study was approved by the Ethics Committee of Blinded for peer review, and included the written informed consent of all participants. All research procedures were conducted in accordance with the ethical standards of the Declaration of Helsinki.

### 2.2. ITLN-1 expression in liver cancer tissue and patient prognosis

A total of 149 HCC patients were divided into low- and high-expression groups according to the median of the relative ITLN-1 expression level. The relationship between ITLN-1 expression and clinicopathological parameters, including age, sex, race, tumor stage, TNM stage, tumor grade, and recurrence, was analyzed, and the survival times of patients with different ITLN-1 expression levels were compared. Univariate and multivariate survival analyses were conducted for patients with different ITLN-1 expression levels to determine whether ITLN-1 is an independent prognostic factor for liver cancer.

### 2.3. Real-time fluorescence quantitative polymerase chain reaction

Trizol reagent was used to extract the total RNA from liver cancer tissues and adjacent normal liver tissue. The RNA was reverse-transcribed into cDNA using ReverTra Ace qPCR RT Master Mix (Toyobo, Japan). SYBR Green was used for quantitative PCR using primers purchased from Wuhan Qing Biotechnology Company (China), with β-actin as internal control. Real-time PCR were performed as previously described using following primer pairs: ITLN-1 sense: 5′-AGTGTTGGACTGACAACGGC-3′, ITLN-1 antisense: 5′-TACATCCGGTGACCCTCATTC-3′; β-actin sense: 5′-CATGTACGTTGCTATCCAGGC-3′, β-actin antisense: 5′-CTCCTTAATGTCACGCACGAT-3′. The results of the analysis were normalized using the 2^−ΔΔCT^ method.

### 2.4. Immunohistochemistry (IHC)

A total of 149 pairs of HCC and adjacent tissues were detected for ITLN-1 expression by immunohistochemical staining. Paraffin-embedded HCC and adjacent tissue sections were dewaxed in xylene and hydrated in ethanol. The slices were immersed in 3 % hydrogen peroxide for 10 minutes, and then the slices were boiled in citrate antigen repair solution (pH = 6.5) for 4 minutes. The slices were incubated overnight with primary antibody at 4 °C. Incubated with second antibody for half an hour. After dyeing with 3,3-diaminobenzidine for 10 minutes, it was re-dyed with hematoxylin, dehydrated and sealed. Each slice was evaluated independently by 2 pathologists.

### 2.5. Western blot

Western blotting was performed in 6 pairs of HCC and adjacent tissues. Take 20 μL sample, total protein was extracted from tissues or cells using a frozen radioimmunoassay precipitation assay buffer containing a complete protease inhibitor cocktail (Roche Diagnostics Corp., Indianapolis, IN) (20 mM sodium phosphate, 150 mM NaCl pH 7.4, 1% nonidet *P*-40, 0.1% sodium dodecyl sulfate and 0.5 % deoxycholic acid).Protein in the culture supernatant was concentrated using a 10,000 MWCO spin column (Millipore, Billerica, MA). Protein expression in the lysate or supernatant was quantified using the BCA Protein Assay Kit (Thermo Fisher Scientific, MA) and boiled at 99 °C for 10 minutes. Proteins were separated by sodium dodecyl sulfate-polyacrylamide gel electrophoresis and transferred onto polyvinylidene fluoride membranes (Merck Millipore, Burlington, MA). blocked with 5% skimmed milk or bovine serum albumin in Tris-buffered saline containing Tween-20 at room temperature for 1.5 hours. The membranes were incubated with primary antibodies overnight at 4 °C and before being incubated with appropriate horseradish peroxidase-conjugated secondary antibodies at 1:10,000 dilution for 1 hour at ambient temperature. Signals were developed using ECL chemiluminescence detection reagents (Thermo Fisher Scientific, MA) and quantified using Image Lab software (version 5.2.1, Bio-Rad, Hercules, CA).

### 2.6. Statistical analysis

The data were analyzed using SPSS20.0 and GraphPad Prism software. The chi-squared test was used to determine the correlation between ITLN-1 expression and clinicopathological variables. Differences with *P* < .05 were considered statistically significant. Cumulative survival time was calculated using the Kaplan–Meier method and compared using the logarithmic rank sum test. Cox proportional risk regression analysis was used to analyze the influence of different clinicopathological characteristics and ITLN-1 expression on the survival rate.

## 3. Results

**Figure 2.**
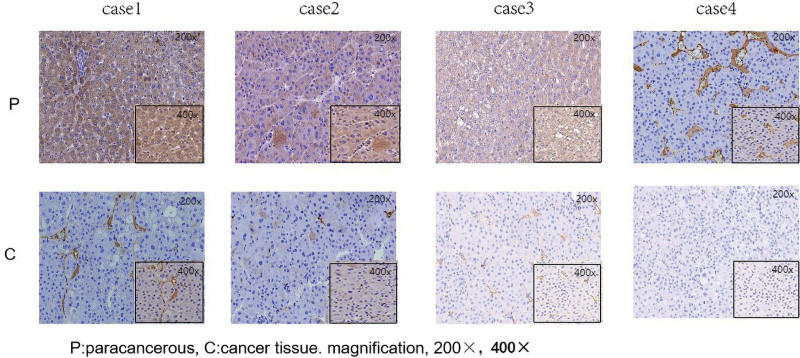
The expression of ITLN-1 in carcinoma and adjacent normal tissues by immunohistochemistry, and ITLN-1 was highly expressed in adjacent tissues.

**Figure 3.**
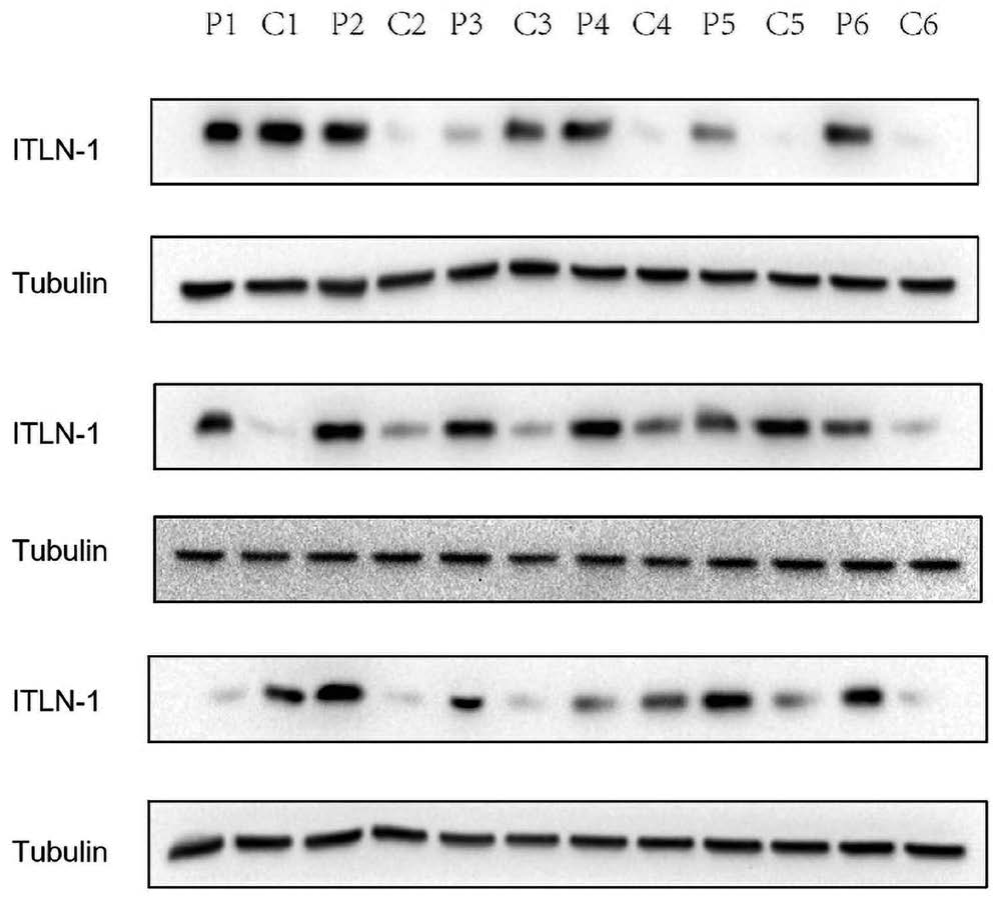
The expression of ITLN-1 in 6 pairs of carcinoma and adjacent normal tissues by Western blot, and ITLN-1 was highly expressed in adjacent tissues.

The clinical characteristics of the 149 HCC patients were summarized in Table 2. The study population included 128 male and 21 female patients with a median age of 47 years (range, 15–79 years). A total of 33 patients had vascular invasion, 59 patients had a maximal tumor diameter ≤ 5 cm, 99 patients had an increased serum AFP concentration, 137 patients were positive for hepatitis B virus surface antigen and 95 patients had grade I or II tumors. Immunohistochemical staining showed that ITLN-1 was highly expressed in adjacent tissues of the same patient, and was lowly expressed in cancer tissues (Fig. 2). Western blot was used to detect the expression of ITLN-1 in 6 pairs of HCC and its adjacent tissues. For Western blot results, the tumor sizes of the 3 pairs of specimens were 2.3 cm, 4.0 cm, and 5.1 cm, respectively, including 1 case at stage T1, 1 case at stage T2, and 1 case at stage T3.The results showed that the expression of ITLN-1 was higher in the adjacent tissues (Fig. 3).

**Table 2:** Correlation between ITLN-1 and clinicopathological variables of HCC.

Variables	Patients, n	ITLN-1 expression		X^2^	*P*
		Low, n (%)	High, n (%)		
Age (years)				0.806	.369
≤45	71	58 (49.6%)	13 (40.6%)		
>45	78	59 (50.4%)	19 (59.4%)		
Gender				0.75	.387
Male	128	99 (84.6%)	29 (90.6%)		
Female	21	18 (15.4%)	3 (9.4%)		
AFP				0.097	.755
Positive	50	10 (31.3%)	40 (34.2%)		
Negative	99	22 (68.7%)	77 (65.8%)		
Tumor status				3.64	.056
T1/T2	95	70 (59.8%)	25 (78.1%)		
T3/T4	54	47 (40.2%)	7 (21.9%)		
Tumor size (cm)				6.665	.010
< 5	59	40 (34.2%)	19 (59.4%)		
≥ 5	90	77 (65.8%)	13 (40.6%)		
Tumor differentiation				6.112	.013
I	25	15 (12.8%)	10 (31.3%)		
II–III	124	102 (87.2%)	22 (68.7%)		
BCLC stage				0.194	.66
0-A	51	39 (33.3%)	12 (37.5%)		
B-C	98	78 (66.7%)	20 (62.5%)		
Vascular invasion				37.385	<.001
Positive	33	26 (51.0%)	7 (7.2%)		
Negative	116	25 (49.0%)	91 (92.8%)		
Tumor nodules				2.028	.154
Single	106	80 (68.4%)	26 (81.3%)		
Multiple	43	37 (31.6%)	6 (18.7%)		
HBV				0.096	.757
Positive	137	108 (92.3%)	29 (90.6%)		
Negative	12	9 (7.7%)	3 (9.4%)		
HBV copies				1.899	.168
Positive	99	81 (69.2%)	18 (56.3%)		
Negative	50	36 (30.8%)	14 (43.7%)		
Capsule				50.495	<.001
Incomplete	57	49 (67.1%)	8 (10.5%)		
Complete	92	24 (32.9%)	68 (89.5%)		
Recurrence				41.582	<.001
No	47	12 (12.8%)	35 (63.6%)		
Yes	102	82 (87.2%)	20 (36.4%)		
Cirrhosis				46.104	<.001
I	63	19 (20.7%)	44 (77.2%)		
II–III	86	73 (79.3%)	13 (22.8%)		

AFP = alpha-fetoprotein; BCLC = Barcelona Clinic Liver Cancer; ITLN-1 = intelectin-1.

**Figure 4.**
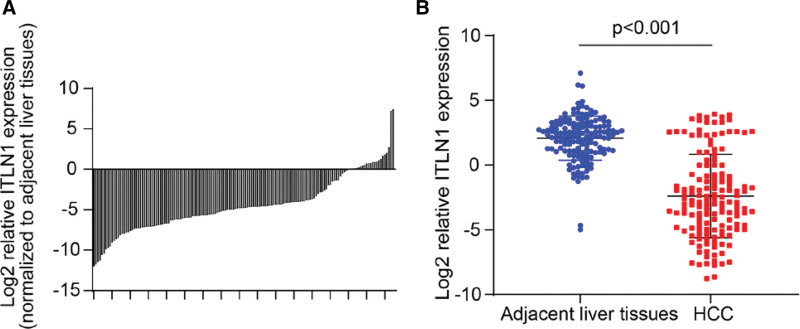
A/B: there were 117 cases (78.5%) with low expression of ITLN-1 in tumor tissues.

In order to verify the expression of ITLN-1 mRNA in liver cancer tissues, we quantified the expression of ITLN-1 in 149 liver cancer tissues and matched non-tumor tissues by RT-PCR and IHC. For IHC results, The tumor sizes of the 4 samples were 2.3 cm, 2.0 cm, 4.3 cm, and 5.1 cm, respectively, including 2 cases at T1 stage, 1 case at T2 stage, and 1 case at T3 stage. According to the expression level of ITLN-1, compared with GAPDH, the number of cycles of ITLN1 was as follows: minimum + 12.8 and maximum + 0.39, and its cutoff value + 10 was calculated. Thus, ITLN-1 was divided into high expression group and low expression group. The results showed that there were 117 cases (78.5%) with low expression of ITLN-1 in tumor tissues (Fig. 4 A/B), and there were 22 (14.7%) cases of low expression of ITLN-1 in normal tissues. The expression of ITLN-1 mRNA in tumor tissues was significantly lower than in adjacent non-tumor tissues (*P* < .01). This result was consistent with our previous gene chip results. According to the chi-squared test, the expression of ITLN-1 was significantly correlated with tumor size (*P* < .001), degree of pathological differentiation (*P* < .001), vascular invasion (*P* < .001), degree of cirrhosis (*P* < .001), capsule status (*P* < .001) and recurrence (*P* < .001), but not with gender, age, AFP, tumor stage, or hepatitis B surface antigen.

**Figure 5.**
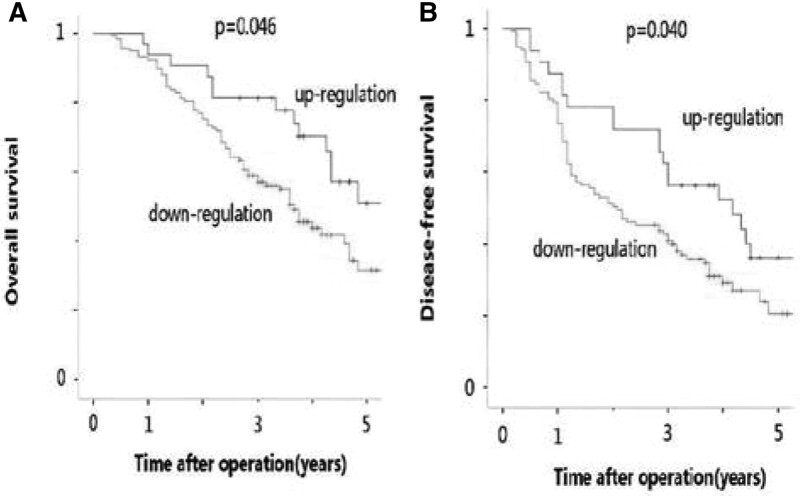
(A) ITLN-1 expression was an independent prognostic factor for OS, and the OS rates of HCC patients with low expression of ITLN-1 after hepatectomy were 92.3, 57.0 and 31.5%, which was significantly lower than those of HCC patients with high expression of ITLN-1. (B) ITLN-1 expression was also an independent prognostic factor for DFS, and the DFS rates after hepatectomy were 79.5, 40.9 and 20.5% in HCC patients with low expression of ITLN-1, which was significantly lower than the corresponding values of 87.5%, 62.5%, and 36.2% in the group with high expression of ITLN-1.

As shown in Table 3, Kaplan–Meier analysis showed that the OS (*P* < .001) and DFS (*P* < .001) of patients with high tumor expression of ITLN-1 were significantly better than those of patients with low ITLN-1 expression in tumor tissues. Univariate Cox regression analysis showed that the risk of death and recurrence in HCC patients increased with tumor size, capsule status, degree of cirrhosis, vascular invasion, and low expression of ITLN-1. Multivariate analysis confirmed that ITLN-1 expression was an independent prognostic factor for OS, and the OS rates of HCC patients with low expression of ITLN-1 after hepatectomy were 92.3, 57.0 and 31.5%, which was significantly lower than those of HCC patients with high expression of ITLN-1 (HR, 0.513; 95% CI, 0.283–0.929 *P* = .046) (Table 4 and Fig. 5A). In addition, ITLN-1 expression was also an independent prognostic factor for DFS, and the DFS rates after hepatectomy were 79.5%, 40.9%, and 20.5% in HCC patients with low expression of ITLN-1, which was significantly lower than the corresponding values of 87.5%, 62.5%, and 36.2% in the group with high expression of ITLN-1 (HR, 0.483; 95% CI, 0.268–0.871; *P* = .04) (Table 5 and Fig. 5B).

**Table 3:** Association between ITLN-1 mRNA expression, clinical parameters and disease-free/overall survival.

Clinical characteristics	Patients, n	Disease-free survival			Overall survival		
		Median, months	95% CI	*P*-value	Median, months	95% CI	*P*-value
ITLN-1 mRNA				<.001			<.001
Low	117	45	43.897–46.103		48	45.138–50.862	
High	32	54	45.340–62.660		56	50.228–61.772	
Age, years				.457			.444
≤45	71	45	43.107–46.893		50	40.700–59.300	
>45	78	47	42.871–51.129		51	43.861–58.139	
Gender				.296			.512
Male	128	47	44.821–49.179		50	43.917–56.083	
Female	21	39	36.863–41.137		57		
AFP				.442			.853
Positive	50	48	40.949–55.051		48	39.905–56.095	
Negative	99	45	43.537–46.463		52	45.744–58.256	
Tumor status				.391			.163
T1/T2	95	47	44.067–49.933		51	44.246–57.751	
T3/T4	54	42	34.962–49.038		45	40.597–49.403	
Tumor size				.038			.026
< 5	59	48	44.054–51.946		54	46.340–61.660	
≥ 5	90	42	37.888–49.112		45	40.370–51.630	
Tumor differentiation				.138			.320
I	25	48	42.846–53.154		49	40.517–57.483	
II–III	124	48	42.871–53.129		52	45.400–58.600	
BCLC stage				.157			.141
0-A	51	52	45.600–58.400		56	49.342–62.658	
B–C	98	45	39.316–50.684		47	44.804–49.196	
Tumor nodules				.870			.923
Single	106	46	43.793–48.207		50	43.197–56.803	
Multiple	43	48	30.606–65.394		54	39.934–68.066	
HBV				.699			.334
Positive	137	46	43.885–48.115		56	32.227–79.773	
Negative	12	45	40.188–49.812		49	44.342–53.658	
Capsule				.015			.023
Incomplete	57	43	42.124–45.876		46	43.862–52.138	
Complete	92	48	45.126–50.874		51	43.722–58.278	
Cirrhosis				.030			.045
I	63	54	46.769–61.231		57	49.058–64.942	
II–III	86	45	43.081–46.919		47	44.788–49.212	
Vascular invasion				.032			.028
Positive	33	**42**	37.650–50.350		44	39.762–50.238	
Negative	116	47	44.882–49.118		52	45.910–58.090	
Recurrence				.024			.013
No	47	47	44.183–49.817		47	44.481–49.519	
Yes	102	43	40.011–49.989		62	50.759–73.241	

AFP = alpha-fetoprotein, BCLC = Barcelona Clinic Liver Cancer, CI = confidence interval;ITLN-1,Intelectin-1;

**Table 4:** Cox multivariate proportional hazards model of independent predictors on disease-free.

Parameter	HR (95% CI)	*P*
Tumor size, cm (<5 vs ≥5 cm)	0.716 (0.432–1.186)	.194
Tumor capsule (yes vs no)	1.105 (0.619–1.971)	.737
Cirrhosis (mild vs moderate-severe)	0.512 (0.303–0.863)	.012
Vascular invasion	1.091 (0.587–2.028)	.784
Recurrence (yes vs no)	0.743 (0.426–1.297)	.743
ITLN-1 mRNA (low vs high)	0.483 (0.268–0.871)	.040

CI = confidence interval, HR = hazard ratio.

**Table 5:** Cox multivariate proportional hazards model of independent predictors on overall survival.

Parameter	HR (95% CI)	*P*
Tumor size, cm (<5 vs ≥5 cm)	0.690 (0.407–1.168)	.167
Tumor capsule (yes vs no)	0.918 (0.520–1.620)	.767
Cirrhosis (mild vs moderate-severe)	0.606 (0.358–1.024)	.061
Vascular invasion	0.781 (0.418–1.458)	.438
Recurrence (yes vs no)	2.114 (1.245–3.591)	.006
ITLN-1 mRNA (low vs high)	0.513 (0.283–0.929)	.046

HR = hazard ratio; CI = confidence interval.

## 4. Discussion

Despite improvements in early diagnosis and combined treatment, HCC remains one of the deadliest cancers in the world.^[16]^ Understanding the molecular mechanisms that drive the pathogenesis of liver cancer will contribute to the development of new therapies.^[17]^ At present, surgical resection, radiofrequency ablation, hepatic arterial chemoembolization, liver transplantation and targeted drugs are still the main treatment methods for HCC.^[18–20]^ Despite advances in the treatment of liver cancer, the recurrence rate remains high and the prognosis remains poor. Currently, AFP is the only serum marker for the diagnosis of HCC, but the sensitivity is low (25–65%), especially for the detection of early HCC.^[21–23]^ AFP alone is not ideal for the monitoring or diagnosis of HCC.^[24]^ Therefore, there is an urgent need to explore new molecular markers, and verify if they can be used for the early detection of the disease, the prediction of the occurrence and development of the disease, as well as the recurrence of the tumor.

Human intestinal lectin-1 (“intestinal lectin-1’’, also known as ITLN-1 and Oentin-1) is a 34 kDa secretory protein originally identified for its ability to bind to the galactofuran unit in the carbohydrate chain of bacterial cell walls.^[25]^ Therefore, it is considered to play an important role in innate immunity against bacteria. Subsequently, it has been reported that ITLN-1 is highly expressed in visceral adipose tissue, especially in interstitial vascular cells, and is less expressed in adipocytes.^[26]^ In vitro studies have shown that ITLN-1 increases insulin signal transduction by activating protein kinase B and enhances insulin-stimulated glucose transport in isolated human adipocytes. Similarly to adiponectin, plasma levels of ITLN-1, are reduced in obese patients, and are associated with insulin resistance.^[26–28]^

Additionally, a series of reports suggested a potential role of ITLN-1 in cancer development.^[7–9,11,12,29,30]^ Li et al reported that ITLN-1 increases the level of hepatic nuclear factor 4α (HNF4α), thereby inhibiting the nuclear localization and transcriptional activity of β-catenin in gastric cancer cells.^[12]^ They also found that high ITLN-1 levels were significantly associated with better outcomes in patients with gastric cancer. Similarly, Kim et al identified ITLN-1 as a favorable prognostic marker in stage IV colorectal cancer.^[29]^ These findings suggest that ITLN-1 acts as a tumor suppressor in gastrointestinal cancers. However, higher ITLN-1 expression was found to be associated with higher risk of colorectal cancer in a prospective cohort study.^[31]^ In addition, ITLN-1 expression was elevated in prostate cancer and decreased in kidney and breast cancer.^[32–34]^ These controversial findings may reflect different roles of ITLN-1 in the development of different cancer types. Recent studies have shown that ITLN1 can affect growth-related pathways regulated by Akt, and ITLN1 can promote Akt phosphorylation in adipocytes, osteoblasts, and mesenchymal cells.^[35,36]^ We found that inhibiting Akt in osteoblasts reduces the proliferative effect of ITLN1 on osteoblasts, suggesting that ITLN1 may function through the Akt pathway. In mesenchymal cells, ITLN1 can also promote the proliferation of Akt regulation, resist oxidative stress, and promote the secretion of angiogenic factors in mesenchymal cells.^[35]^ This suggests that ITLN1 may play an important role in tumor mesenchymal stem cells and thus affect tumor growth.

In this study, we found that the expression of ITLN-1 was significantly lower in HCC tissues than in adjacent non-tumor tissues. Low expression of ITLN-1 was associated with a larger tumor volume, poor pathological differentiation, vascular invasion, incomplete capsule and occurrence of cirrhosis. To our best knowledge, this is the first study to investigate the relationship between ITLN-1 and the clinical outcomes of HCC patients. The mRNA level of ITLN-1 in tumor tissues was significantly lower than in adjacent normal liver tissues (*P* < .05). Low expression of ITLN-1 was found to be associated with recurrence and vascular invasion in HCC patients. In addition, the expression of ITLN-1 was significantly correlated with OS and DFS, suggesting that loss of ITLN-1 expression may be associated with the progression of HCC and can be used as a potential independent prognostic indicator in HCC patients. Taken together, the results of this study indicate that ITLN-1 may inhibit the occurrence and development of HCC, thus playing the role of a tumor suppressor gene. However, the relationship between ITLN-1 and the pathogenesis of HCC, as well as whether ITLN-1 can be used as a therapeutic target for liver cancer still needs further study. According to our results, low expression of ITLN-1 may be a worrisome sign and a higher priority should be given to the patient. In such cases, early intervention is recommended to reduce tumor recurrence and improve the survival. In conclusion, our data suggest that ITLN-1 is a predictor of the survival of HCC patients, and that ITLN-1 may serve as a novel marker for predicting the recurrence and clinical outcomes of HCC.

Although the clinical significance of ITLN-1 in HCC was revealed, several limitations still exist in our study. Firstly, it is a retrospective cohort in a single center with limited patients. Therefore, patients from multicentric cohort are necessary to confirm our findings. Although some studies have suggested that ERK signaling mediates the function of ITLN-1, the detailed mechanisms by which ITLN-1 exerts these effects are unclear and therefore require further study. The reason for the reduced expression of ITLN-1 in cancer is unknown. Further studies are needed to elucidate the molecular mechanisms by which ITLN-1 is dysregulated in HCC.

## 5. Conclusions

The loss of ITLN-1 expression plays an important role in the proliferation, occurrence and development of liver cancer, and is an independent prognostic factor for OS and disease-free survival. To our best knowledge, this is the first report of a correlation between ITLN-1 expression and the clinical outcomes of HCC patients. These results suggest that the expression level of ITLN-1 may be an important factor affecting the survival of patients, and it can be used as a prognostic indicator for HCC patients. Nevertheless, larger and prospective multicenter studies are needed to confirm these findings.

## Author contributions

**Conceptualization:** Erlei Zhang.

**Data curation:** Hai-Su Tao, Tong Yuan.

**Formal analysis:** Hai-Su Tao, Tong Yuan.

**Funding acquisition:** Erlei Zhang.

**Methodology:** Jiang Li.

**Project administration:** Jiang Li.

**Writing – original draft:** Jiang Li.

**Writing – review & editing:** Zhi-Yong Huang.
